# Coping with Job Loss and Reemployment: A Two-Wave Study

**DOI:** 10.1007/s10869-014-9380-7

**Published:** 2014-09-26

**Authors:** Erica Solove, Gwenith G. Fisher, Kurt Kraiger

**Affiliations:** Department of Psychology, Colorado State University, Campus Box 1876, Fort Collins, CO 80523-1876 USA

**Keywords:** Job loss, Reemployment, Coping resources, Coping strategies

## Abstract

Using a national sample, this study investigated the effects of unemployed workers’ coping resources and coping strategy use on reemployment after a three-month period. Based on previous research, it was expected that (1) three types of coping resources (self-esteem, social support, and financial resources) would be positively related to problem-focused coping with job loss, (2) coping resources would be negatively related to emotion-focused coping with job loss, (3) problem-focused coping would be positively related to reemployment, (4) problem-focused coping would be more strongly related to reemployment than emotion-focused coping, and (5) coping strategies would mediate the relationship between the availability of coping resources and obtaining reemployment. Results provided support for the direct effects of coping resources (self-esteem, social support, and, to some extent, financial resources) on coping strategies, and a direct effect of problem-focused coping on reemployment 3 months later. Self-esteem and social support were each indirectly related to subsequent employment status, mediated by problem-focused coping. In other words, individuals with higher levels of self-esteem and social support were not only more likely to engage in problem-focused coping, but having a higher level of self-esteem and social support was also associated with a higher likelihood of being reemployed three months later. Findings are pertinent for the design of more effective interventions that mitigate adverse effects of unemployment and facilitate a successful return to the workforce.

## Introduction

Understanding the experience of job loss holds considerable significance for unemployed workers. This is a particularly important and salient issue in light of the enormous spike in unemployment rates that occurred recently during the Great Recession beginning in late 2008. The Great Recession was the worst economic recession in the world in almost 100 years (Borbely 2009). Although unemployment rates have recovered to some extent since this global economic crisis, as of this writing, global unemployment is still highly prevalent (International Labour Organization 2014). For example, in January of 2014, the number of unemployed persons in the United States totaled 10.2 million, or 6.6 % of the working population (Bureau of Labor Statistics 2014). Global unemployment increased by five million people in 2013 compared to 2012, now totaling almost 202 million people worldwide (International Labour Organization 2014).

Job loss is considered an extremely stressful and jarring life event in which paid employment is involuntarily removed from an individual (Latack et al. 1995). Over the course of their working lives, many workers face the dire experience of job loss. Early unemployment research focused on an array of negative physical and psychological consequences associated with experiencing job loss, and demonstrated that unemployment has a consistent negative effect on physical and psychological well-being beyond obvious financial hardship (Feather 1990; Kessler et al. 1989; McKee-Ryan et al. 2005; Wanberg 1995). Although making truly causal inferences is as yet unfounded, research has shown that unemployment is consistently associated with negative outcomes such as heart disease, depression, anxiety, alcohol abuse, mortality, and even suicide (Jin et al. 1995; Wanberg, 2012).

## Coping with Job Loss

Unemployment research has been conducted steadily since the Great Depression. During this time, such research has revealed considerable individual differences in responses to job loss (Leana and Feldman 1992). To explain this variability in responses, researchers have focused more recently on the impact of coping during the stressful experience of this involuntary event (Gowan et al. 1999; Latack et al. 1995; Leana and Feldman 1992). Although many different conceptualizations of coping exist (Latack and Havlovic 1992), Lazarus and Folkman (1984) defined coping broadly as behavioral and cognitive efforts used to manage an appraised stressor. Some general functions of coping are to gather information about the demands of the stressor, reduce tension, and restore a state of equilibrium (Lazarus and Folkman 1984). In other words, coping refers to what a person actually thinks or does to manage a stressor and minimize strain (Lazarus 1991). Coping is a situation-specific phenomenon (Latack et al. 1995) and is often used as an umbrella term to describe behaviors, cognitions, or strategies employed in a difficult situation (Schwarzer and Schwarzer 1996). Many studies have demonstrated large individual variability in coping during stressful life situations (Lazarus and Folkman 1984), as coping with hardships involves complex person–environment interactions (Schwarzer and Schwarzer 1996).

Coping with the particular stressor of job loss has been defined as a person’s constantly changing behavioral and cognitive efforts to manage internal or external demands that are associated with unemployment and are appraised as surpassing the resources possessed by the individual (Folkman et al. 1986). Despite a large body of job loss and unemployment literature, the coping processes specifically following job loss have received relatively little attention in existing research (McKee-Ryan et al. 2005). Latack et al. (1995) claimed that the majority of job loss research has used generic coping models, without addressing the specific, complex mechanisms, through which coping processes are created, which subsequently affect crucial outcomes for particular stressors. The current study will consider coping in terms of the specific stressor of involuntary job loss, as coping with involuntary job loss is quite different than coping with other life stressors such as divorce or death. Although many studies demonstrate that individuals experience fear, anger, grief, and sadness when coping with involuntary job loss, the loss of a job is perceived as more reversible than the loss of a marriage or a loved one (Blustein et al. 2013; Wanberg 2012).

One possible antecedent to coping strategies is coping resources. In their unemployment meta-analysis, McKee-Ryan et al. (2005) reported a relationship among coping resources, coping strategies, and well-being during unemployment. Coping resources are defined as a set of internal (e.g., self esteem) and external (e.g., financial resources, social support) factors that a person may use to cope with involuntary job loss (Latack et al. 1995). However, McKee-Ryan et al. emphasized that more research is needed to understand the impact of coping resources and coping strategies on reemployment. Specifically, the researchers stated a need for research examining how different forms of job loss coping may be differentially beneficial and the mediating or moderating relationships between coping resources, coping strategies, and reemployment. More recently, Wanberg (2012) explicated the need for robust models examining the relative importance of variables associated with reemployment success, including coping resources and strategies. The present study responds to these calls for better models and more research, thereby making a substantive contribution to the literature. By examining the process by which job loss coping resources and coping strategies are related to achieving reemployment in a path model, the current study directly addresses these gaps. Practically, investigating the coping resources and coping strategies that relate to achieving reemployment can inform the design of more effective interventions that mitigate adverse effects of unemployment and facilitate a successful return to the workforce (Blustein et al. 2013; Wanberg 2012).

## Theoretical Framework

Latack et al. (1995) proposed an integrative process model of coping with job loss, integrating Lazarus and Folkman’s (1984) seminal coping theory with Edwards’ (1992) control theory. Building upon inadequately generic, superficial past models of coping with job loss, this integrative process model explicates the specific mechanisms and processes of coping with job loss and their subsequent impact on relevant outcomes (Latack et al. 1995). By understanding how job loss affects coping, one can propose and test factors that buffer the negative outcomes of job loss. The integrative process model posits that job loss disrupts the equilibrium between an individual’s desired and perceived existing states. Engaging in a coping response alters this disequilibrium, subsequently resulting in a feedback loop for a new, modified coping response. According to this model, the ultimate goal of coping with job loss is to reduce the discrepancy so that equilibrium is restored in various disrupted life facets, specifically psychological, physiological, social, and economic facets (Edwards 1992).

### Coping Resources

According to the integrative process model of coping with job loss, an individual’s discrepancy appraisal is affected by the availability of coping resources, which serve to mitigate the harmful impact of involuntary job loss (Latack et al. 1995). Past job loss studies have defined coping resources in various ways. For example, Gowan et al. (1999) conceptualized coping resources as education, financial resources, and social support. Vinokur and Schul (2002) examined coping resources such as mastery, job-search self-efficacy, and job-search motivation, and psychological vulnerabilities such as financial strain and elevated depressive symptoms.

Coping resources can have both direct and indirect effects on recovery from job loss. Directly, the negative effects of job loss can be lessened or buffered through the application of coping resources. Indirectly, coping resources can trigger the use of cognitive strategies or increase the effectiveness of such strategies, leading to recovery from job loss. Prior empirical research has provided support for both proposed paths. For example, Vinokur and Schul (2002) found that job-search motivation (a coping resource) had a direct positive impact on reemployment 6 and 12 months later, and financial strain aided reemployment by increasing job-search motivation and job-search intensity but also inhibited reemployment by increasing depressive symptoms. Gowan et al. (1999) found that social support (another coping resource) was positively related to all coping strategies (distancing from job loss, involvement in job-search activities, and non-work activities), whereas education and financial resources were only related to job-search and non-work activities. Lastly, McKee-Ryan et al.’s (2005) meta-analysis of psychological and physical well-being during unemployment included personality, social support, financial resources, and ability to structure one’s time as coping resources. They found that financial strain was negatively related and social support was positively related to psychological health of unemployed individuals.

The present study examined three types of resources congruent with coping resources proposed by Latack et al. (1995): self-esteem (personal), the availability of social support from friends and family (social), and the availability of financial resources (financial). Self-esteem is defined as a person’s overall evaluation or appraisal of his or her own worth (Rosenberg 1965). Unemployed individuals possessing high self-esteem use this resource to drive the intensity of their job search, which is related to obtaining reemployment (Prussia et al. 2001). Social support is defined as helping relationships within a social network (Caplan et al. 1975). Social support has been related to diminished stressful effects of job loss, as social support encourages unemployed individuals to maintain optimism and increases their readiness to search for new employment options (Gowan et al. 1999; Zikic and Klehe 2006). Lastly, financial resources are defined as the level of perceived economic hardship (Wanberg et al. 2002). The availability of financial resources prevents unemployed individuals from experiencing immediate financial crisis caused by struggling to pay day-to-day bills (Gowan et al. 1999). While a financial cushion has been associated with increased psychological health during unemployment (McKee-Ryan et al. 2005), lack of financial resources has been associated with faster reemployment (Kanfer et al. 2001). Instead of waiting for the right job, unemployed individuals who lack financial resources presumably experience more pressure to jump at the first opportunity to earn a paycheck (Wanberg et al. 2002). Temporally, coping resources are more plentiful immediately following job loss but tend to lessen over the duration of unemployment (Latack et al. 1995).

### Coping Strategies

According to Latack et al.’s (1995) job loss coping model, individuals reacting to involuntary job loss engage in coping strategies in an attempt to reduce their disequilibrium-induced stress. Coping strategies have been conceptualized in several different ways in the literature, including a focus on particular populations, focus on specific stressful situations, focus on coping over time, or an overarching general focus (Schwarzer and Schwarzer 1996). However, job loss coping strategies have most often been conceptualized as either problem-focused or emotion-focused coping strategies (Hanisch 1999). *Problem*-*focused coping* involves deliberately mitigating or eliminating the stressor by objectively and analytically taking action ideally by addressing the cause of the problem (in this case, unemployment). Examples include acquiring additional marketable skills by seeking training, actively searching for new job opportunities, or relocating to another city with better job prospects (Leana et al. 1998). In contrast, *emotion*-*focused coping* involves easing the emotionally distressing feelings caused by the stressor through strategies like avoidance, distancing, or minimizing the problem. Examples of emotion-focused coping with job loss include expressing frustration or sadness about not having a job, downplaying the seriousness of job loss, or engaging in community activism to aid others in the community who are also unemployed (Leana et al. 1998).

In response to a stressor, the choice of using problem-focused and emotion-focused coping strategies is complex and dependent on numerous factors. In general, problem-focused coping is more likely to occur when conditions of the stressor are appraised as possible to change by taking action, whereas emotion-focused coping is more likely to occur when conditions are appraised as more difficult to change. However, neither coping style is necessarily superior in all contexts, as the perceived utility of each depends on various personal and situational factors (Latack 1986; Lazarus and Folkman 1984; Wanberg 1997). For example, it may be more advantageous for an individual to engage in specific strategies to find alternative employment (i.e., problem-focused coping) than to vent frustrations and express disappointment about being unemployed (i.e., emotion-focused coping), as an emotion-focused coping style may exacerbate the negative feelings and stress associated with losing one’s job.

Availability of coping resources has been positively related to problem-focused coping and negatively related to emotion-focused coping in past research. For example, Kinicki et al. (2000) observed positive relationships between coping resources (self-esteem, life satisfaction, and social support) and problem-focused coping strategies and negative relationships between coping resources and emotion-focused coping strategies among unemployed workers. In addition, Wanberg et al. (1996) demonstrated that job-seeking social support was positively associated with problem-focused coping and negatively associated with emotion-focused coping among job-seeking individuals following a layoff. In turn, problem-focused coping (as opposed to emotion-focused coping) has been consistently related to obtaining reemployment (Hanisch 1999; Kinicki et al. 2000; Leana et al. 1998, Wanberg 1997). For example, Leana et al. (1998) found that unemployed individuals who used more problem-focused coping were significantly more likely to achieve reemployment. Kinicki et al. (2000) found a negative relationship between emotion-focused coping of unemployed workers and the quality of reemployment. The present study builds upon prior research by examining the ways in which these three key components (coping resources, coping strategies, and reemployment) are related in a single model, and testing whether coping strategies serve as a mediating mechanism between coping resources and reemployment, which previously has not been done. Specifically, we examine the direct effects of coping resources on coping strategies and reemployment (a response to job loss), and whether coping strategies mediate relationships between coping resources and reemployment. Thus, the present study contributes to the existing literature by examining coping with job loss as a process over time, wherein coping strategies serve as a mechanism for obtaining reemployment.

### Present Study

**Fig. 1:**
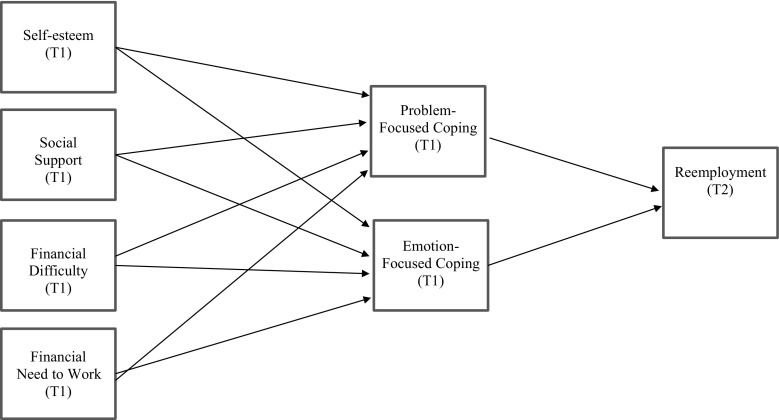
Hypothesized model of coping with job loss and subsequent employment status

Using the aforementioned conceptualization of coping resources and coping strategies, we developed a path model in which coping strategies mediate the relationship between coping resources and reemployment (see Fig. 1). Specifically, Latack et al.’s (1995) integrative process model of coping with job loss states that coping resources have a direct effect on coping strategies. According to this model, availability of coping resources (i.e. self-esteem, social support, and financial resources) directly affects an individual’s appraisal of the gap between his or her current unemployed state and a future desired employed state. Unemployed individuals with ample self-esteem believe in their abilities to secure a new job, leading to problem-focused coping activities directly focused on obtaining a new job. Unemployed individuals lacking self-esteem possess diminished faith in their capabilities to obtain reemployment, leading to emotion-focused coping activities that provide a form of escape. Possessing social support provides unemployed individuals with many pragmatic benefits for securing a new job, including networking, moral support, and soliciting job leads, resume writing help, or interviewing tips. Without the considerable emotional boost that social support brings, unemployed individuals are more likely to compensate by coping through emotion-focused strategies involving withdrawal or avoidance. Finally, having a financial cushion allows unemployed individuals to optimally position themselves for a new job through problem-focused coping activities like networking or traveling to job interviews, lessening panic about basic survival and affording day-to-day expenses. The added worry and limited opportunities associated with inadequate financial resources causes unemployed individuals to engage in more emotion-focused coping behaviors. Thus, an abundance of coping resources narrows the appraisal gap and triggers problem-focused coping aimed at pragmatically securing reemployment. Conversely, lacking these resources widens the appraisal gap and results in more emotion-focused coping strategies primarily aimed at easing distress. These assertions, grounded in Latack et al.’s (1995) model, are congruent with Kinicki et al.’s (2000) findings demonstrating that coping resources were positively related to problem-focused coping with job loss and negatively related to emotion-focused coping with job loss. Based on this theoretical framework and past empirical findings, we hypothesize the following:

#### **Hypotheses 1 & 2**

Coping resources will be positively related to problem-focused coping (H1), and coping resources will be negatively related to emotion-focused coping (H2).

Drawing from Latack et al.’s (1995) model, problem-focused coping entails behaviors aimed at relinquishing the stressful situation of involuntary joblessness. Because finding work can be challenging, problem-focused coping strategies are important because they increase pragmatic job-search activities. Problem-focused coping involves engaging in behaviors that increase the probability of finding a job, therefore affecting reemployment. In contrast, emotion-focused coping entails behaviors aimed at relinquishing the feelings and responses caused by the stressful situation. Although emotion-focused coping behaviors may alleviate the emotional distress caused by job loss, these behaviors do not affect persistence that leads to positive reemployment outcomes. Accordingly, based on both theory and prior findings (Hanisch 1999; Leana et al. 1998, Wanberg 1997), we hypothesize the following:

#### **Hypotheses 3 & 4**

Problem-focused-coping strategies used at Time 1 will be positively related to reemployment status at Time 2 (H3). In other words, individuals who report more use of problem-focused coping will be more likely to be reemployed 3 months later than those who report less use of problem-focused coping. Further, problem-focused coping will be more strongly related to reemployment than emotion-focused coping (H4).

Finally, a recognized need exists for research explicating the mechanisms by which coping resources and coping strategies affect reemployment success (McKee-Ryan et al. 2005; Wanberg 2012). Latack et al.’s (1995) model posited a relationship between coping resources and coping strategies in which diminished or bountiful coping resources can influence the coping strategy used. According to this model, coping resources affect discrepancy appraisal because they serve as a repository of support sources that can be used to shape coping strategies, ultimately leading to the attainment of reemployment. Thus, it is expected that individuals with higher levels of coping resources (self-esteem, social support, and financial resources) will have greater capabilities to use problem-focused coping, and congruent with Hypotheses 3 and 4, will have a higher likelihood of achieving reemployment success 3 months later. Consequently, we expect that coping resources will be indirectly related to reemployment with coping strategies as a mediator, and hypothesize the following:

#### **Hypothesis 5**

Coping strategies will mediate the relationship between the availability of coping resources at Time 1 and reemployment status at Time 2 (H5).

The hypothesized relationships were tested using a field sample of unemployed workers with data collected at two points in time, 3 months apart.

## Method

### Participants and Procedure

314 unemployed workers were included in this study. Participants were recruited online from Meetup.com and LinkedIn.com job loss groups through a posted description of the study and link to the survey. Meetup.com is a Web site which facilitates the creation and organization of local groups in communities throughout the world, and currently has 15.9 million members and over 142,000 local groups meeting about various topics worldwide. LinkedIn.com is a Web site that facilitates social networking among business professionals worldwide, and its over 277 million members can also create virtual groups about various topics.

Meetup and LinkedIn Group members who chose to participate were instructed to click on a link to the web-based survey that was created and administered using Qualtrics online survey software. After clicking the survey link, participants were directed to a page detailing the study’s purpose and informed consent. This page also explained that only individuals who involuntarily lost their last jobs and were actively seeking reemployment were eligible to participate. By choosing to click past this initial page, informed consent was obtained. During recruitment, participants were told that they would not receive compensation for completing the initial survey, but that they would receive $20 for participation in the subsequent follow-up survey. Most respondents (91.7 %) indicated that they were interested in participating in a follow-up survey.

Data were collected in two waves. Participants in the first wave were 314 unemployed adults who had lost their jobs involuntarily, and were actively seeking reemployment. On average, participants had been unemployed for a year and a half (*M* = 1.53 years, SD = 1.08). 58.6 % of the sample was female. The majority of participants were of middle-age or older adults, with a mean age of *M* = 51.9 (SD = 9.0) years; 10.8 % under age 40, 71 % between 40 and 59 years old, and 18.2 % age 60 or older. The majority of the sample (83.8 %) identified themselves as White/Caucasian, 9.6 % identified themselves as Black/African-American, 3.8 % identified themselves as Hispanic, 2.2 % identified themselves as Asian, 1.6 % identified themselves as Native American, and 2.5 % identified themselves as some other race/ethnicity (not specified).^1^ Most participants (81.2 %) had at least a college degree; one third (33.4 %) earned a graduate degree.

A follow-up web survey was conducted 3 months later, using the same procedure as the initial survey. This survey included all first wave measures as well as a measure of reemployment status. 123 participants completed the follow-up survey, for a follow-up response rate of 39.2 %. Non-respondents to the follow-up survey did not differ significantly on any of the demographic characteristics or study variables compared to those who completed both surveys (*p* > .10). Unfortunately, employment status at the time of the follow-up survey was unknown among study participants in Time 1 who did not complete the follow-up survey.

### Measures

#### Coping Resources

Coping resources represent a collection of aids that a person can use to reduce the negative effects of a stressful situation. For the instance of job loss, researchers have generally identified three types of coping resources: personal (self-esteem), social (social support), and financial (financial resources) (Latack et al. 1995; McKee-Ryan et al. 2005). The measurement of these three types of coping resources (self-esteem, social support, and financial resources) was as follows.

*Self*-*Esteem* Self-esteem was measured at Time 1 with the widely-used ten-item Rosenberg Self-Esteem Scale (Rosenberg, 1965). A sample item from this scale is, *“I feel that I have a number of good qualities.”* Responses were obtained on a four-point Likert scale anchored from *strongly agree* (4) to *strongly disagree* (1). The internal consistency of the self-esteem scale in this study was *α* = .88.

*Social Support* Social support was measured at Time 1 with the Social Support Scale developed by Caplan et al. (1975) as modified by Gowan et al. (1999). Two scales were used to operationalize the construct of social support: support from relatives and support from friends. Four items were used to measure each scale. A sample item for the variable support from relatives is, *“Since your job ended, how much can your closest relatives be relied on when things get tough?”* Correspondingly, a sample item for the variable support from friends is, *“Since your job ended, how much can your closest friends be relied on when things get tough?”* Responses were obtained on a five-point Likert scale anchored from *don’t have any such person* (0) to *very much* (4). Responses were averaged to generate a global measure of social support. The internal consistency of the social support scale in this study was *α* = .85.

*Financial Resources* Financial resources were measured with two items assessing perceived economic hardship from Wanberg et al. (2002). The first item is, *“How difficult is it for you to live on your total household income (including your unemployment benefits and income from others persons) right now?”* Responses for this item were on a three-point Likert scale anchored from *not at all difficult* (1) to *extremely difficult* (3). The second item is, *“How important is it for you, financially, to find a job within the next two months?”* Responses for this item were obtained on a three-point Likert scale anchored from *not at all important* (1) to *very important* (3). The correlation between the two items is *r* = .41, *p* < .05. Given the moderate correlation between these two items, they were not combined to reflect a single indicator of financial resources, but were both used as indicators in the tested path model.

#### Coping with Job Loss

Coping with job loss was measured at Time 1 with 11 items from Kinicki and Latack’s (1990) Coping with Job Loss Scale (CWJLS). The CWJLS is a widely-used instrument (see Lai and Chan 2002; Kinicki et al. 2000; McKee-Ryan et al. 2005; Wanberg 1997) and the first standardized scale developed to measure coping specifically related to involuntary job loss. Items in this measure indicate either “control” or “escape” coping, which are synonymous with problem-focused coping and emotion-focused coping, respectively (see Latack et al. 1995). In the present study, the CWJLS items were divided into a six-item “problem-focused coping” scale (*α* = .88) that indicates the extent to which individuals engage in a problem-focused proactive strategy in which unemployed individuals take control of the situation, and a five-item “emotion-focused coping” scale (*α* = .81) that reflects an emotion-focused avoidance of actions or thoughts related to the job loss. Consistent with prior research (Lai and Chan 2002), a subset of items from the CWJLS was used to create two distinct coping factors. Sample items for the problem-focused coping scale are *“Focus my time and energy on job search activities“* and *“Talk with people who can help me find a job.” * Sample items for the emotion-focused coping scale are, *“Tell myself that there are more important things in life than having a job”* and *“Tell myself that time usually takes care of situations like this.”* Responses were given using a five-point Likert scale anchored from *hardly ever do this* (1) to *almost always do this* (5). The measure’s content validitywas established with subject matter experts, and construct validity was established with factor analysis and by experimentally examining the process of coping with job loss over time (Kinicki and Latack 1990).

#### Reemployment Status

At Time 2 (i.e., 3 months later), respondents were also asked to report their current employment status. Specifically, respondents selected one of three options: (1) *Unemployed,* (2) *Employed, but in a job they did not want*, or (3) *Employed in a job they wanted*. The distinction between (2) and (3) was made to consider satisfaction with a new job in addition to basic acquisition of a new job in measures of reemployment status (McKee-Ryan et al. 2009; see also Wanberg et al. 2002). However, for the purposes of the present study in which we examined continued unemployment versus reemployment, a dichotomous variable was created by combining response options (2) and (3).

#### Demographics

The demographic variables of age, gender, race, marital status, number of dependents, date of job loss, and education were also collected.

### Statistical Analysis

We used path analysis in M*plus* version 7 (Muthén and Muthén 1998–2012) to assess the relations among coping resources and coping strategies at Time 1 and employment status (reemployed vs. still unemployed) at Time 2. We estimated all equations simultaneously using a single model (see Fig. 1) as described by Preacher and Hayes (2008). We used bias-corrected bootstrapping procedures (5,000 draws) to estimate indirect effects, standard errors, and statistical significance (Preacher and Hayes 2008). We controlled for respondents’ age and length of unemployment when examining all the paths in the model. We controlled for age because older workers in our sample reported being less likely to be employed 3 months later. Controlling for age is also supported by Johnson and Butrica’s (2012) finding that unemployment duration increases considerably for adults 49 and older. Duration of unemployment measured at Time 2 was included as a control variable. Prior research has generally statistically controlled for duration of unemployment (McKee-Ryan et al. 2005), as the duration of unemployment is related to the way in which individuals react to the stressor of involuntary job loss in terms of coping resources and coping strategies (Kanfer et al. 2001). More generally, coping resources tend to diminish over time during the duration of unemployment (Latack et al. 1995). Kinicki et al. (2000) found that financial strain increased and that unemployed workers displayed more coping behaviors as unemployment persisted over time.

Although other demographic variables were measured in the present study (e.g., gender and education), they were not included as control variables in the analysis because they were unrelated to any of the study variables (*p* > .05). This is consistent with recommendations by Carlson and Wu (2012), who advocated for conservative use of control variables and omitting them when they exhibit nonsignificant correlations with study outcomes.

In the statistical analysis, the MISSING command was used. Although data were complete for the Time 1 measures, use of this command imputes values when estimating paths for data missing at Time 2. We evaluated the validity of this approach by separately conducting the analysis using listwise deletion for a total sample size of *n* = 123. Results were very consistent with those obtained using the larger sample. Results reported in the next section are based on the sample of *n* = 314.

## Results

Table 1 presents descriptive statistics and correlations for all study variables. The coping resources of self-esteem and social support were positively related to one another, negatively related to financial difficulty, and positively related to both problem-focused and emotion-focused coping strategies. Age was positively related to duration of unemployment and negatively related to reemployment 3 months later. Duration of unemployment was negatively related to self-esteem.

**Table 1:** Descriptive statistics and bivariate correlations for study variables

		*M*	SD	1	2	3	4	5	6	7	8
1	Age (in years)	51.85	9.00								
2	Years of unemployment	1.53	1.64	.19^**^							
3	Self-esteem (T1)	3.51	.56	.12	−.16^**^						
4	Social support (T1)	3.64	.83	.00	−.12	.28^*^					
5	Financial difficulty (T1)	2.35	.64	−.06	.08	−.20^**^	−.17^**^				
6	Financial need to work (T1)	2.67	.59	−.11^*^	.04	−.12	−.08	.41^**^			
7	Problem-focused coping (T1)	3.99	.77	.11*	−.09	.34^**^	.20^**^	.03	.13^*^		
8	Emotion-focused coping (T1)	2.78	.98	−.01	−.06	.34^**^	.36^**^	−.28^**^	−.18^*^	.22^**^	
9	Reemployment (T2)	.44	.50	−.19^*^	.04	−.06	.07	−.02	.08	.19^*^	−.01

*N* = 314, except for Reemployment (*N* = 123). * *p* < .05, ** *p* < .01. T1 = Time 1 (first survey administration). T2 = Time 2 (second survey administration, 3 months later)

**Fig. 2:**
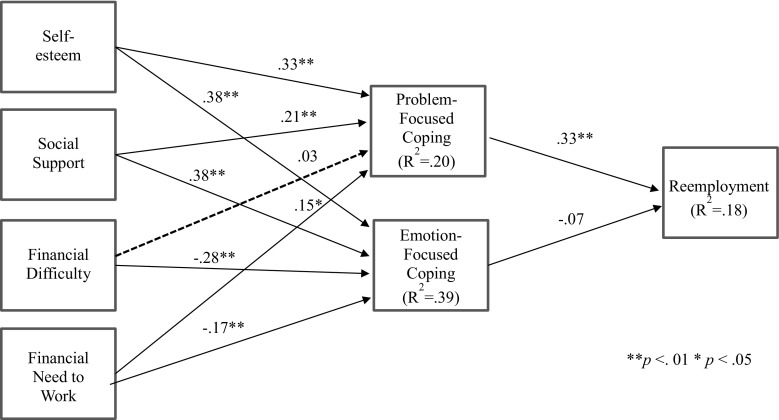
Path model results: coping with job loss and subsequent employment status

Results of the mediation model that was tested are shown in Table 2 and Fig. 2. Results generally supported Hypothesis 1, which stated that coping resources are positively related to problem-focused coping. Self-esteem, social support, and needing to work for financial reasons were related to problem-focused coping strategies, although experiencing financial difficulty was unrelated to problem-focused coping. Support was mixed regarding Hypothesis 2, that coping resources are negatively related to emotion-focused coping. Self-esteem and social support were positively related to emotion-focused coping, but financial resources were negatively related to emotion-focused coping. Social support was more strongly related to emotion-focused coping than to problem-focused coping. Results supported Hypotheses 3 and 4, as problem-focused-coping strategies used at Time 1 were positively related to subsequent employment status, but emotion-focused coping strategies were unrelated to reemployment. Controlling for age and duration of unemployment at Time 2, results supported the hypothesized mediation model for problem-focused coping, such that problem-focused coping mediated the relationship between resources (self-esteem, social support) and subsequent reemployment. Results demonstrated significant indirect effects of self-esteem and social support on reemployment. However, emotion-focused coping did not mediate the relationship between coping resources and unemployment.

**Table 2:** Direct and indirect effects results

Predictor variable	Age	Length of unemployment	Problem-focused coping	Emotion-focused coping	Reemployment
Direct effects					
Self-esteem	.16**	−.19**	.33**	.38**	
Social support	.03	−.13*	.21**	.38**	
Financial difficulty	−.07	.10	.03	−.28**	
Financial need to work	−.12*	.06	.15*	−.17**	
Problem-focused coping	.10	−.04			.33**
Emotion-focused coping	−.11*	.10			−.07
Reemployment	−.32**	.14			
Indirect effects via problem-focused coping					
Self-esteem					.11**
Social support					.07*
Financial difficulty					.01
Financial need to work					.05
Indirect Effects via Emotion-Focused Coping					
Self-esteem					−.03
Social support					−.03
Financial difficulty					.02
Financial need to work					.01
Total *R* ^2^			.20**	.39**	.18**

*N* = 314. * *p* < .05 ** *p* < .01. Problem- and emotion-focused coping were measured at T1; Reemployment was measured at T2. Standardized estimates (path coefficients) presented for direct and indirect effects. All analyses control for age and years of unemployment

## Discussion

The purpose of this study was to examine a mediation model in which coping resources are related to reemployment through the mediating mechanism of coping strategies. We hypothesized that coping resources would be positively related to problem-focused coping (H1), negatively related to emotion-focused coping (H2), and positively related to later reemployment status (H3). We further hypothesized that problem-focused coping would more strongly predict reemployment status than would emotion-focused coping (H4), and coping strategies would mediate the relationship between coping resources and reemployment outcomes (H5). In general, our hypotheses were supported. Results provided support for the direct effects of coping resources (self-esteem, social support, and, to some extent, financial resources) on coping strategies, and a direct effect of problem-focused coping on reemployment 3 months later. Self-esteem and social support were each indirectly related to subsequent employment status, mediated by problem-focused coping. In other words, individuals with higher levels of self-esteem and social support were not only more likely to engage in problem-focused coping, but having a higher level of self-esteem and social support at Time 1 was also associated with a higher likelihood of being reemployed at Time 2. Coping resources were related to emotion-focused coping. Consistent with prior research (e.g., Hanisch 1999; Leana et al. 1998), emotion-focused coping was unrelated to reemployment.

The results of this study provide further clarity for past empirical findings examining the relationship between job loss coping resources, coping strategies, and reemployment success. The present study addresses Wanberg’s (2012) call for research to investigate the relative importance of variables associated with reemployment (and the interactions among these variables) in relation to reemployment success. In addition, the present study directly answers McKee-Ryan et al.’s (2005) call for research elucidating the distinctive impact of job loss coping strategies on unemployment outcomes and investigating the mediating or moderating relationships between coping resources, coping strategies, and reemployment. While these prior studies examined the aforementioned direct effects, the present study modeled the direct and indirect relationships between these variables by testing a cohesive mediation model of the relationships among coping resources, coping strategies, and reemployment. This is an important contribution because it provides insight into the unemployment coping process by articulating resources and specific coping strategies that are associated with more positive reemployment outcomes. Furthermore, the present study exhibited the relative importance of problem-focused coping (as opposed to emotion-focused coping) in relation to reemployment.

Understanding how coping strategies mediate the relationship between coping resources and reemployment outcomes is an important step in the development of a full model explicating the ways in which individuals cope with, and work through, job loss. Although there is no established model to understand the dynamic nature of the coping process throughout the course of unemployment (Kinicki et al. 2000), empirical evidence suggests that reactions to job loss and accompanying coping strategies occur in stages and fluctuate with a feedback loop of discrepancy reduction between actual state (unemployed) and desired state (reemployed). For example, shock or anger initially experienced soon after the event of job loss may be related to increased emotion-focused coping. Job devaluation is a common example of an emotion-focused coping strategy at this early stage, as it involves cognitively convincing oneself that there are many more important things in life other than having a job (Kinicki and Latack 1990). In contrast, if an individual begins to tap his or her available social networks after some time has passed and starts to engage in intense job-search behaviors, then this individual would be displaying problem-focused coping strategies centered on the goal of obtaining a new job (Latack et al. 1995; Wanberg et al. 2002). Considering the overall lack of longitudinal study designs in unemployment research (Wanberg et al. 2002), the present study demonstrates the significant impact of problem-focused coping strategies on eventual reemployment success.

Greater coping resources, such as self-esteem and social support, were positively related to the use of problem-focused strategies for coping with job loss. These findings are consistent with prior research that has found that availability of personal and situational coping resources are positively related to problem-focused coping and negatively related to emotion-focused coping (Kinicki et al. 2000). The present study’s findings were mixed regarding the hypothesized negative relationship between all three coping resources and emotion-focused coping. Specifically, while financial resources were negatively related to emotion-focused coping, social support and self-esteem were positively related to emotion-focused coping. Gowan et al. (1999) found similar results in which the coping resource of social support was positively related to both problem-focused and emotion-focused coping strategies used by individuals following involuntary job loss. Aligned with their proposed model, the study by Gowan et al. (1999) found that the greater the availability of social support, the greater the likelihood that individuals engaged in both job seeking activities like networking (problem-focused coping) and reevaluated the situation as less stressful due to the emotional support of friends and family (emotion-focused coping). Although Gowan et al. (1999) did not include self-esteem as a coping resource, it is reasonable to presume that self-esteem may have a similar dual effect on coping strategies in which higher self-esteem would be related to greater confidence in executing job seeking behaviors and an increased ability to emotionally distance oneself from the distress of involuntary job loss due to a heightened confidence that reemployment is indeed achievable. Ultimately, the results between coping resources and emotion-focused coping may be more complex than we hypothesized, and further research is necessary to better understand these relationships.

### Limitations

This study has a few important limitations that are worth noting. First, the sample in this study was likely not fully representative of unemployed workers across the U.S., taking into account many demographic characteristics (e.g., age, occupation, and socioeconomic status). Participants in this study were recruited through online groups for unemployed individuals. This sample recruitment strategy may have introduced several potential sources of bias. First, study participants had access to a computer and the Internet, leading to a bias against unemployed individuals who do not have access to these resources or do not know how to use these resources. Although technology proficiency has become increasingly expected in most professions (Baruch 2006), it is incorrect to assume that individuals who have computer and Internet proficiency represent workers of all professions or demographic groups (Couper et al. 2007). Secondly, because participants were recruited online through job loss support resources, it is likely that our sample may have differed from the population of unemployed workers in important and meaningful ways. Our participants were actively seeking unemployment resources, networking, and social support, so they may have differed from the population in regards to their coping strategies, mental and physical health, or other relevant individual differences such as conscientiousness.

The results obtained in the present study may be due to the especially resilient, pragmatic nature of the sample, as all participants had the initiative to access and utilize online unemployment resources. In addition, although there were no significant demographic differences between participants in the study’s two waves, it is possible that participants who found employment 3 months later were less likely to participate in the second survey because the topic was less relevant to them, the $20 incentive was less attractive, and/or they had less spare time. Consequently, our study may have included a smaller proportion of reemployed individuals in our sample at Time 2, possibly attenuating the relationships due to restriction of range in the dependent variable. Despite these noteworthy limitations of the sample, the present study utilized a unique recruitment strategy, as past job loss studies have ordinarily recruited through state workforce centers (e.g., Wanberg et al. 2002). Moreover, the recruitment technique utilized in the present study resulted in a national sample. Acknowledging the limitations of this study’s sample, future research should replicate the present study with a larger, more heterogeneous sample of unemployed individuals rather than only those from online support groups.

Although the methodology was strengthened on account of collecting data across two distinct points in time 3 months apart, both the independent variables (coping resources) and mediator variables (coping strategies), were measured concurrently at Time 1. This was not an optimal temporal design for testing mediation (Ployhart and Ward 2011). Future longitudinal research is warranted to examine the relations between the independent/exogenous variables and the mediators.

Finally, common method bias may be another limitation of this study (Podsakoff et al. 2003), as all data were obtained through self-report Likert-type surveys from the same source. Common method bias is problematic because using the same method to measure various constructs can either inflate or deflate the observed relationships between the measured constructs. Although it is possible that the findings in the present study may reflect common method bias, the methodology was strengthened on account of collecting data across two distinct points in time 3 months apart. However, the measurement of both the independent variables (coping resources) and mediator variables (coping strategies) at Time 1 was not the optimal temporal design (Ployhart and Ward 2011). Second, not all of the study variables were correlated or were all paths in the model significant, which suggests that not all relations between variables were necessarily inflated due to using a common method.

### Future Directions

Many opportunities exist for future research in this area. Job loss research must expand the conceptualization of unemployment to account for two workforce trends: underemployment and bridge employment. Underemployment occurs when a worker is employed, but in a job considered below his or her full working capability (McKee-Ryan et al. 2009). Similarly, future job loss research must consider the changing nature of retirement by incorporating bridge employment literature. Bridge employment, a growing phenomenon, is defined as a longitudinal workforce participation process which takes place following a person’s retirement from full-time work but before the person’s complete withdrawal from the workforce (Wang and Shultz 2010). This intermediary stage in the retirement process is becoming increasingly common, and may be motivated by workers’ desire to gradually adjust to retirement life, financial necessity, or health reasons (Wang and Shultz 2010). Integrating underemployment and bridge employment literature into job loss studies will contribute to understanding the complexities experienced by the unemployed segment of the workforce, as several calls for future research exist to understand the increasingly dynamic nature of employment across the life-span and quality of reemployment as a superior criterion (McKee-Ryan et al. 2009; Wang and Shultz 2010). For example, additional research is needed to examine the extent to which workers seek opportunities to remain in the labor force and do so by obtaining bridge employment, but find themselves underemployed rather than working in a job that better matches their knowledge, skills, and abilities. Furthermore, such research should examine outcomes associated with underemployment and bridge employment. Workers who are underemployed or working in bridge jobs may experience work in very different ways compared to workers in other phases of their careers.

Related to the changing nature of retirement, it is interesting to speculate how our findings would generalize across age groups, as the majority of respondents in the present study were middle-aged or older. Although we controlled for age in our analyses, the issue of coping with unemployment in an aging workforce is a burgeoning area of research as the aging “Baby Boomer” generation has caused an influx of older workers (Hedge et al. 2006). It is estimated that by 2015, one in five US workers will be of age 55 or older (Avery et al. 2007). Thus, the need to understand the aging process and the differential experiences that accompany aging in the workforce is becoming an increasingly pressing matter (Griffiths 2003). Existing research suggests that vulnerability to job loss is heightened later in life, due to an increased difficulty in finding new opportunities after suffering from involuntary job loss (Hedge et al. 2006; Ito and Brotheridge 2006; Ng and Feldman 2009).

In relation to coping, Leana and Feldman (1992) found that older individuals coping with job loss appraised their situations as less reversible than did younger individuals, which aligns with Johnson and Butrica’s (2012) finding that older adults (49 and older) experienced longer durations of unemployment. While a considerable body of research has examined differences in coping with various stressors across the life-span (Aldwin et al. 2007; Boerner and Jopp 2007; Riediger et al. 2006), there is a notable dearth of studies focusing on the relationship between age and coping specifically with job loss (Hanisch 1999). However, prominent life-span coping theories and supporting empirical studies suggest that the use of emotion-focused coping strategies generally increase later in life (Baltes and Baltes 1990; Brandtstädter and Renner 1990; Carstensen 1993; Heckhausen and Schulz 1995; Riediger et al. 2006), which may be adaptive for older individuals facing additional limitations. Thus, a fruitful area for future research would be to examine the differential use of coping strategies from an aging perspective, and the ensuing impact on coping resources and reemployment.

In addition, more longitudinal research is needed in the area of job loss. The process of coping with job loss is dynamic, and fluctuates over time based on factors internal and external to the unemployed individual (Wanberg et al. 2002). These changes are impossible to capture with cross-sectional study designs, and difficult to study over two time points. Gathering data at three or more time points spanning months or years would enable researchers to more effectively examine the job loss experience, reemployment success, and health and well-being outcomes, as well as take into account external environment factors such as the macroeconomic climate and labor market (Wanberg et al. 2002).

## Conclusion

In conclusion, this study demonstrated that unemployed workers who used problem-focused coping strategies were more likely to be employed 3 months later compared to those who utilized emotion-focused coping, and coping resources were related to reemployment through the mediating mechanism of coping strategies. Our results extend prior empirical findings regarding coping resources and coping strategies by modeling the mechanisms by which reemployment is more likely to occur. In addition, the relation between coping strategies and reemployment success was examined using a two-wave design, which is often lacking in unemployment research that has primarily relied upon cross-sectional research designs (Wanberg et al. 2002). Future research is warranted to further clarify the relations between coping resources, coping strategies, and reemployment status. However, results suggest that coping strategies are an important part of the unemployment experience and obtaining reemployment. Job loss support groups, workforce centers, and other resources for the unemployed should teach unemployed workers problem-focused coping strategies.

## Footnotes

1 Percentages for participant race/ethnicity add up to greater than 100 % because participants could select multiple options.
